# Acute Inhibition of Adipose Triglyceride Lipase by NG497 Dysregulates Insulin and Glucagon Secretion From Human Islets

**DOI:** 10.1210/endocr/bqaf090

**Published:** 2025-05-12

**Authors:** Lucy B Kim, Siming Liu, Syreine Richtsmeier, Michał Górniak, Anamika Vikram, Yumi Imai

**Affiliations:** Department of Internal Medicine, Carver College of Medicine, University of Iowa, Iowa City, IA 52242, USA; Fraternal Order of Eagles Diabetes Research Center, University of Iowa, Iowa City, IA 52242, USA; Department of Internal Medicine, Carver College of Medicine, University of Iowa, Iowa City, IA 52242, USA; Fraternal Order of Eagles Diabetes Research Center, University of Iowa, Iowa City, IA 52242, USA; Iowa City Veterans Affairs Medical Center, Iowa City, IA 52246, USA; Department of Internal Medicine, Carver College of Medicine, University of Iowa, Iowa City, IA 52242, USA; Fraternal Order of Eagles Diabetes Research Center, University of Iowa, Iowa City, IA 52242, USA; Department of Internal Medicine, Carver College of Medicine, University of Iowa, Iowa City, IA 52242, USA; Warsaw University of Life Sciences, 02-787 Warsaw, Poland; Department of Internal Medicine, Carver College of Medicine, University of Iowa, Iowa City, IA 52242, USA; Fraternal Order of Eagles Diabetes Research Center, University of Iowa, Iowa City, IA 52242, USA; Department of Internal Medicine, Carver College of Medicine, University of Iowa, Iowa City, IA 52242, USA; Fraternal Order of Eagles Diabetes Research Center, University of Iowa, Iowa City, IA 52242, USA; Iowa City Veterans Affairs Medical Center, Iowa City, IA 52246, USA

**Keywords:** beta cell, alpha cell, lipid droplets, lipolysis

## Abstract

Adipose triglyceride lipase (ATGL), which catalyzes the breakdown of triglycerides in lipid droplets (LDs), plays a critical role in releasing fatty acids to support insulin secretion in pancreatic β cells. Based on genetic downregulation of ATGL in β cells, multiple mechanisms are proposed that acutely or chronically regulate insulin secretion. Currently, the contribution of acute vs chronic mechanisms in the regulation of insulin secretion is unclear. Also, little is known whether ATGL affects α-cell function. Using the human-specific ATGL inhibitor, NG497, this study investigates the impact of acute inhibition of ATGL on hormone secretion from human islets. In addition, morphological differences in LDs were assessed in confocal images of β and α cells. β cells exposed to NG497 overnight showed notable increases in LD size and number under glucose-sufficient culture. The effect of NG497 on LD accumulation in α cells was more prominent under fasting-simulated conditions than glucose-sufficient conditions, pointing toward a critical role for ATGL lipolysis under conditions that stimulate hormone secretion in β and α cells. When exposed to NG497 acutely, human islets reduced glucose-stimulated insulin secretion mildly, particularly first-phase insulin secretion, to an extent somewhat less pronounced than the impacts of chronic ATGL downregulation. Thus, chronic mechanisms may play a predominant role in reducing insulin secretion when ATGL is downregulated. Acute exposure of human islets to NG497 significantly reduced amino acid stimulated glucagon secretion at low glucose concentration, highlighting an important potential role of ATGL lipolysis in promoting hormone secretion acutely from α cells.

Adipose triglyceride lipase (ATGL, encoded by PNPLA2 gene) is a neutral lipase whose major substrates are triglycerides (TG) in lipid droplets (LDs) (1). In pancreatic beta cells, lipolysis mediated by ATGL is stimulated by glucose and supports insulin secretion through multiple mechanisms revealed by studies of β cells after downregulation of ATGL expression (2, 3). Fatty acids (FA) released from LDs can activate the GPR40 cell surface receptor and potentiate glucose-stimulated insulin secretion (GSIS) (2). 1-monoacylglyceride (1-MAG), another product of lipolysis by ATGL, stimulates exocytosis of insulin granules through MUNC13-1 that promotes SNARE complex assembly (4). In addition, a more chronic contribution of ATGL activity for GSIS is proposed. ATGL knockout in mouse β cells impairs mitochondrial function by reducing activity of peroxisome proliferator-activated receptor δ (PPARδ) (5). The impairment of insulin secretion after ATGL downregulation was associated with decreased syntaxin1a (STX1a) protein levels in both human pseudoislets and INS-1 cells, which is attributed to accelerated degradation of STX1a secondary to reduced palmitoylation (6). In contrast to well-established impairment of insulin secretion after genetic downregulation of ATGL in β-cell models, it is unclear the extent by which acute suppression of lipolysis by ATGL impairs insulin secretion. Acute reduction of GSIS was originally reported in rat islets treated with a pan lipase inhibitor orlistat (7), but a subsequent report has not been consistent regarding the impact of orlistat on GSIS (8). Moreover, orlistat is not a specific inhibitor of ATGL leaving acute contribution of ATGL-mediated lipolysis on GSIS in human islets undefined (9). Another question is the role of ATGL in α cells. Preincubation of mouse islets with FA mixture that expands TG store improves subsequent glucagon secretion when glucose is lowered (10). Considering that FA are proposed to support glucagon secretion by providing α cells energy through fatty acid oxidation (10, 11), lipolysis of TG may regulate glucagon secretion by releasing FA for oxidation.

NG497 is the first human-specific small-molecule inhibitor of ATGL without inhibiting multiple lipases including hormone-sensitive lipase, pancreatic lipase, lipoprotein lipase, and hepatic lipase (12). NG497 is not cytotoxic to hepatocytes up to 100 μM. It acutely blocks forskolin-stimulated lipolysis almost completely in human adipocytes at 40 μM and allows us to assess a role of human ATGL through pharmacological inhibition (12). Here, we tested the effect of ATGL inhibition on LD morphometry under different nutrient conditions in human β cells and α cells as ATGL degrades LDs at the surface of LDs. In addition, the acute effect of NG497 on insulin and glucagon secretion was tested by perifusion assay in human islets to determine the extent by which lipolysis acutely impacts human β- and α-cell function.

## Materials and Methods

### Human Islets

An institutional review board at the University of Iowa deemed that human islet experiments are not human studies. Human islets from Integrated Islet Distribution Program or Alberta Diabetes Institutes (Supplementary Table S1 (13)), with reported viability and purity above 80% were cultured overnight at 37 °C and 5% CO_2_ upon arrival for recovery from shipping. Perifusion of intact human islets were performed the next day after shipment. For morphometry of LDs, human islets dispersed by Accutase (SCR005, Millipore Sigma, St Louis, MO) were plated at approximately 35 000 cells/cm^2^ on a confocal dish coated with 50 μg/mL of Col IV (C5533 from Sigma) as reported (14), and cultured in neuronal medium (minimum essential medium containing 1× glutaMAX [Gibco], 5% fetal bovine serum, 1 mM sodium pyruvate, 10 mM HEPES, and 1× B-27 supplement [Gibco]) described by Phelps et al (15) for 3 days. Glucose concentration of neuronal medium is 11 mM unless specified otherwise. BSA concentration of neuronal medium measured by ELISA (RRID:AB_3675434) was 0.133 mM. For some experiments, islet cells were incubated with neuronal medium containing 2.8 mM glucose and 0.13 mM oleic acid plus 0.27 mM palmitic acid loading (LoG + FA, 3:0 molar ratio of FA mixture:BSA) for overnight. Molar ratio is reported as BSA binds FA and determines the availability of unbound FA to islet cells (16). Human pseudoislets treated with lentivirus expressing shRNA against ATGL and scramble control were previously validated and reported (6).

### Morphometry of LDs

Human islets were incubated in neuronal medium containing 3 μM Bodipy 558/568 C12 (Bodipy C12, ThermoFisher, Waltham, MA) with vehicle (DMSO at 0.05% [v/v]) or 40 μM NG497 (80 mM stock in DMSO, Focus Biomolecules, Plymouth Meeting, PA) at 37 °C at 5% CO_2_ for the last 16 hours of culture. After fixation, cells were immunostained with insulin (INS; rabbit anti-INS antibody, RRID:AB_3673135, at 1:300) or glucagon (GCG, by mouse anti-GCG antibody, RRID:AB_259852, at 1:600) to visualize β and α cells, respectively. Images of the cells were captured by a Zeiss 980 microscope to acquire z-stack images via a 63× oil lens at an interval of 0.19 μm. A set of 5 consecutive slices of the image with the largest footprint of cells were selected from each stack. The size and number of LDs were measured as published (14). The area of beta cells and alpha cells were also obtained to normalize data applying the method used for the measurement of LDs area above.

### Measurement of LD Degradation

To label LDs with Bodipy C12, human islets were incubated in 11 mM of glucose neuronal medium containing 3 μM Bodipy C12 at 37 °C at 5% CO_2_ for the last 16 hours of culture as previously except for omitting DMSO or NG497. Then, medium was changed to fresh neuronal medium with 20 μM triacsin C plus 0.05% (v/v) DMSO or 40 μM NG497 and continued culture. The first set of cells treated with triacsin C plus DMSO were fixed at 30 minutes (baseline), the second set of cells treated with triacsin C plus DMSO at 2.5 hours (DMSO chase), and the third set of cells treated with triacsin C plus NG497 at 2.5 hours (NG497 chase). Areas of LDs in fixed cells were measured as published (14) and corrected for insulin positive area as above. As triacsin C blocks synthesis of new LDs, the reduction of LD area after chase period over baseline represents the degradation of LDs in β cells during the chase period (17).

### Perifusion of Islets

BioRep Perifusion System (BioRep Technologies, Miami Lakes, FL) was used to perifuse human pseudoislets as published (18). For the measurement of insulin secretion, islets were perifused by Krebs-Ringer bicarbonate buffer (KRB) consisting of 120 mM NaCl, 4.8 mM KCl, 2.5 mM CaCl_2_, 1.2 mM MgCl_2_, 24 mM NaHCO_3_, 10 mM HEPES, and 0.25% BSA with addition of 2.8 mM glucose for 48 minutes followed by KRB containing 16.7 mM glucose or 30 mM KCl plus 2.8 mM glucose for indicated time. To measure insulin secretion in the presence of FA, 0.25% BSA was replaced with 0.16 mM palmitic acid and 0.08 mM oleic acids conjugated with 0.6% FA free BSA (2.7:0 molar ratio of FA:BSA) from time 0. For the measurement of glucagon secretion, islets were perifused by KRB containing 3.3 mM glucose for 48 minutes followed by 1 mM glucose plus amino acid mixture (2 mM each of glutamine, alanine, and arginine as published elsewhere (19, 20)), followed by 7 mM glucose plus amino acid mixture for indicated time. Human islets are exposed to amino acid mixture at low glucose first to combine two stimuli of glucagon secretion (21). Of note, stimulating human islets with amino acids at 7 mM glucose first makes subsequent response to switching to 1 mM glucose less well defined for the peak secretion (Supplementary Fig. S1 blue line, first peak vs second peak (22)), and difference between glucose concentration was seen at the second phase of glucagon secretion (Supplementary Fig. S1 after time 80 minutes (22)). Either vehicle (0.05% [v/v] DMSO) or 40 μM NG497 (80 mM stock in DMSO) was added from time 0 for all perifusion. Total insulin and glucagon contents were obtained from islets incubated overnight at 4 °C in RIPA buffer (R0278, Sigma) containing protease inhibitors. Insulin was measured using STELLUX Chemiluminescent Human Insulin ELISA (RRID:AB_2894946). Glucagon was measured using glucagon ELISA kit from Crystal Chem (RRID:AB_2884901).

### Statistical Analysis

Data are presented as mean ± standard error of mean (SEM) unless otherwise stated in the figure legends. Differences of numeric parameters between 2 groups were assessed with Student *t*-tests or Mann-Whitney test using Prism 10 (GraphPad, La Jolla, CA) as indicated. Multiple group comparisons used 1-way ANOVA with post hoc as indicated. A *P* < .05 was considered significant.

## Results

### Human β and α Cells Accumulate LDs Avidly After ATGL Inhibition at the Conditions That Stimulate Their Hormone Secretion

**Figure 1.**
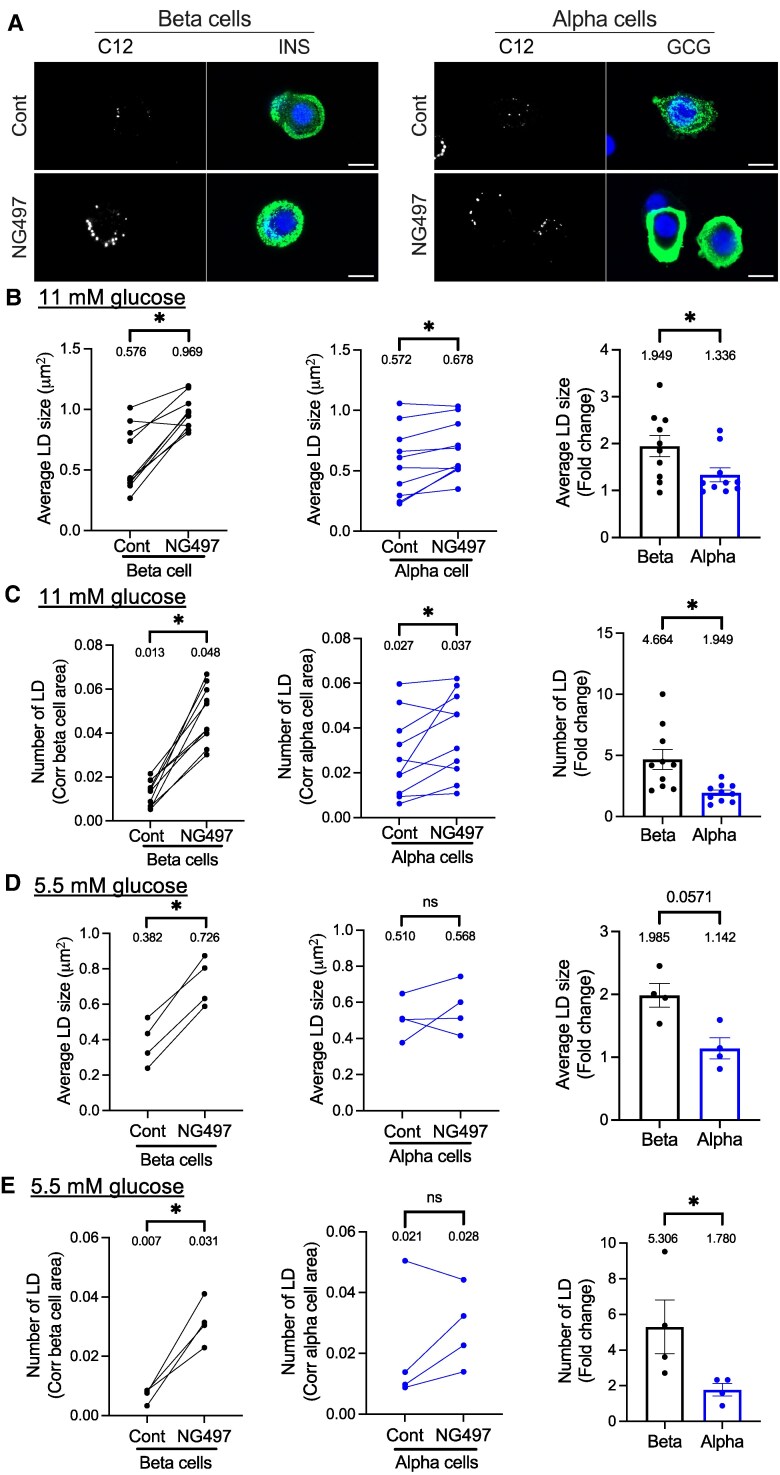
NG497 promotes LD accumulation more prominently in human β cells than α cells under 11 and 5.5 mM glucose. (A) Representative confocal images of human β and α cells cultured in 11 mM glucose medium with overnight addition of vehicle (Cont) or NG497. Cells were stained with DAPI (blue), BODIPY 558/568 C12 (C12, white) to visualize LDs, and INS or GCG (green) to determine β- and α-cell area. Scale bar, 10 μm. (B, C) LD morphometry under 11 mM glucose, n = 10 donors; (D, E) under 5.5 mM glucose, n = 4 donors. (B, D) Average size of LDs and (C, E) average number of LDs corrected for insulin- or glucagon-positive area in Cont and NG497-treated β and α cells. Left and middle panels: Each dot represents 2 donor and values from the same donor are connected by a line. Right: Fold change of NG497-treated cells over Cont. Data are mean ± SEM. *P < .05 by Student *t*-test except for right panels of D, E, for which the Mann-Whitney test was performed.

Balance between the formation and degradation of LDs determines the size and number of LDs in each cell. In β cells, glucose drives both TG synthesis and ATGL-mediated lipolysis of TG (6, 23). Considering TG contents determines LD pool size, we assessed the proportion of LDs being degraded by ATGL under 11 mM glucose medium by performing LD morphometry after NG497 treatment in β and α cells. Average size and number of LDs varied among donors, but they were overall in a similar range for β and α cells cultured in 11 mM glucose medium (Fig. 1A-1C). For both β and α cells, the inhibition of lipolysis overnight by NG497 caused statistically significant increases in the size and number of LDs (Fig. 1A-1C) contributing to the increase in total area occupied by LDs corrected for cell area (Supplementary Fig. S2A (22)). For β cells, the prominent accumulation of LDs by NG497 agrees with the increase of LDs observed in human β cells when ATGL is downregulated by shRNA and indicates that LDs are actively formed and degraded in β cells at 11 mM glucose (6, 23). However, the impact of ATGL inhibition on LD accumulation at 11 mM glucose was less prominent in α cells, shown as lower fold change of LD size, LD number, and total area occupied by LDs compared with β cells (Fig. 1B-1D, right panels, Supplementary Fig. S2A, right panel (22)). At 5.5 mM glucose, NG497 increased size and number of LDs in β cells but not in α cells (Fig. 1D and 1E, Supplementary Fig. S2B (22)). Smaller LD accumulation by NG497 implicates that ATGL activity is lower in α cells than β cells under glucose sufficient conditions.

**Figure 2.**
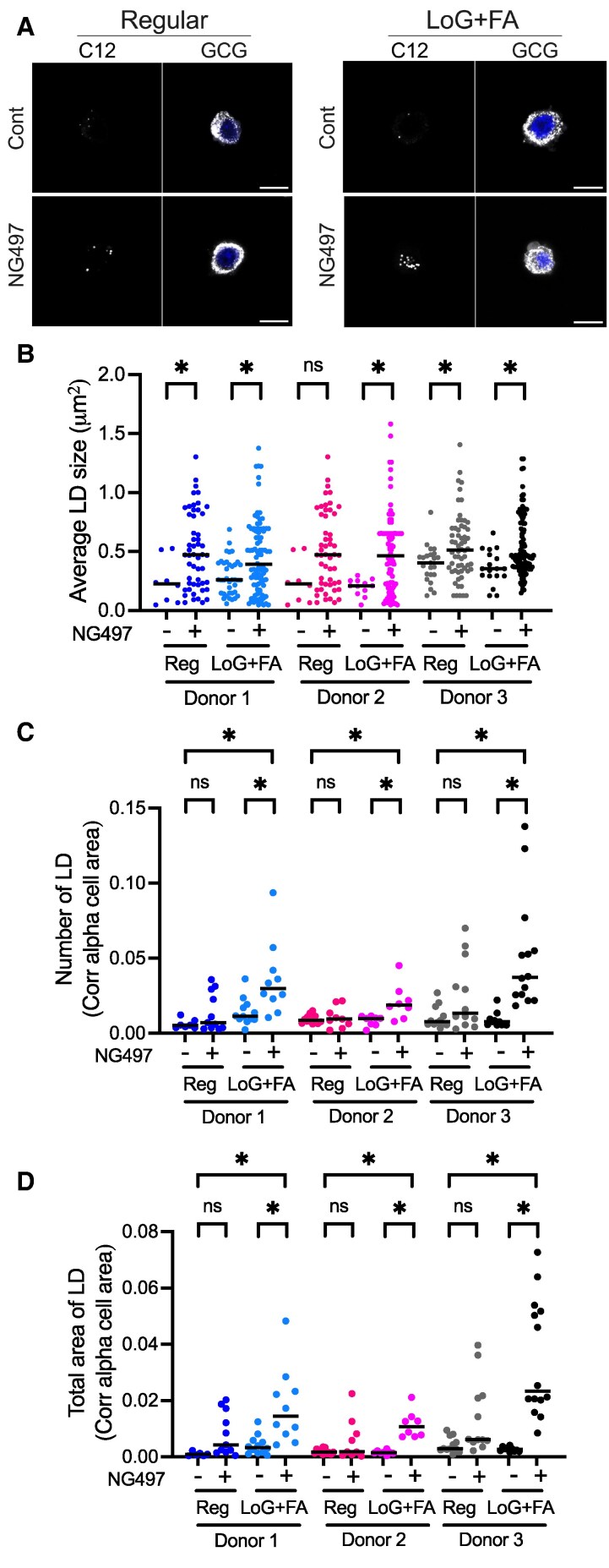
Accumulation of LDs in human α cells increases under low glucose and fatty acid loading. (A) Representative confocal images of α cells cultured in medium containing 11 mM glucose (Reg) or 2.8 mM glucose with 0.13 mM oleic acid plus 0.27 mM palmitic acid loading (LoG + FA) with addition of vehicle (Cont) or NG497 for overnight. α cells were identified via DAPI (blue) and GCG-staining (white), whereas LDs were visualized with BODIPY 558/568 C12 (C12, white). Scale bar, 10 μm. (B) Size distribution of LDs in alpha cells, (C) the number of LDs corrected for glucagon-positive area, and (D) the total LD area occupied per glucagon-positive area for 3 donor islets. Each dot represents an individual LD for (B) and an individual image for (C) and (D). n = 6 to 14 images analyzed. Median is indicated by line. *P < .05; ns, not significant by 1-way ANOVA with Sidak multiple comparison test. Groups compared are indicated by lines at the top.

Considering that glucagon secretion from α cells is more active during fasting when glucose level is low and FA availability is increased (24), we tested whether the disruption of lipolysis affects the degree of LD accumulation more prominently in a culture condition similar to fasting. Human islets with and without NG497 treatment were cultured under 11 mM glucose or 2.8 mM glucose plus 0.13 mM oleic acid and 0.27 mM palmitic acid (LoG + FA) loading overnight before fixation (Fig. 2A). For 3 independent donor islets tested, NG497 increased average LD size to a similar extent both in 11 mM glucose and LoG + FA conditions (Fig. 2B). However, NG497 increased the number of LDs and total LD area per cell area more prominently under LoG + FA conditions than under 11 mM glucose (Fig. 2C and 2D). Thus, LD degradation by ATGL lipolysis in α cells is more active under LoG + FA conditions compared with 11 mM glucose.

### Acute ATGL Inhibition Predominantly Impairs First Phase of Glucose-stimulated Insulin Secretion From Human β Cells

**Figure 3.**
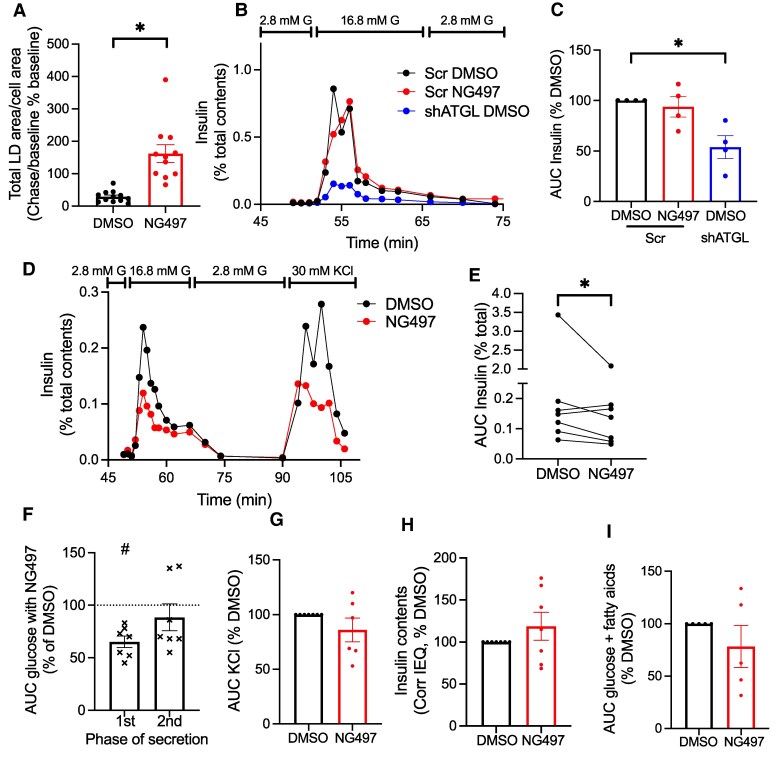
ATGL suppression dysregulates glucose-stimulated insulin secretion from human islets. (A) Total LD area corrected for β cell area of human islet cells exposed to 20 μM triacsin C and 0.05% DMSO or 40 μM NG497 after a 2-hour chase period. Data are expressed as the ratio of post chase/baseline and taking baseline as 100%. Mean ± SEM, n = 11-12 images. Representative of 4 independent experiments. *P < .05 by Student *t* test. (B-C) Human pseudoislets transduced with lentivirus expressing shATGL (shATGL) or scramble control (Scr) were perifused with 2.8 mM glucose KRB containing 0.05% DMSO or 40 μM NG497 from time 0 and exposed to 16.8 mM glucose (G) at the indicated time. Insulin secretion was corrected for insulin contents. (B) A representative profile (donor 11, Supplementary Table S1 (13)), and (C) area under the curve (AUC) of glucose response corrected for insulin contents and expressed by taking the value for Cont DMSO as 100% for each donor islets. n = 4 donor. *P < .05 by repeated measure 1-way ANOVA with Dunnett test. (D-H) Human islets were perifused as in (B) followed by 30 mM KCl for indicated time. Insulin secretion was corrected for insulin contents. (D) A representative profile (donor 5, Supplementary Table S1 (13)). (E) AUC of glucose response. Values from the same donor are connected by a line. (F) First (1st) and second (2nd) phases of glucose-stimulated insulin secretion expressed taking the value for DMSO as 100% for each donor islets. (G) AUC of KCl stimulated insulin secretion corrected for insulin contents and expressed by taking the value for DMSO as 100% for each donor islets. (H) Insulin contents corrected for islet equivalent (IEQ) and expressed taking value for DMSO as 100% for each donor islets. (D-H) n = 7 donors. (I) Human islets were perifused as in (A), except 0.24 mM of FA was added throughout perifusion from time 0 and AUC during 16.8 mM glucose is shown taking the value for DMSO as 100% for each donor islets. n = 5 donors. Data are mean ± SEM. (E)* and F)#; *P* < .05 vs DMSO by paired Student *t*-test.

To assess the impact of acute ATGL inhibition, we first ensured that NG497 blocks lipolysis efficiently after 30 minutes preincubation by measuring lipolysis as the reduction of LD area in β cells in the presence of TG synthesis inhibitor triacsin C (17). As shown in Fig. 3A, total LD area corrected for β-cell area was reduced to 29.3 ± 17.5% of baseline after 2 hours in control but the reduction was completely prevented in the presence of NG497, indicating NG497 is efficacious in suppressing lipolysis.

To compare the impact of acute vs prolonged suppression of ATGL activity on GSIS, GSIS was measured in human pseudoislets in which ATGL was downregulated by shRNA and control pseudoislets acutely exposed to NG497. qPCR confirmed downregulation of ATGL to 27.1 ± 6.3% of scramble control in shATGL-treated pseudoislets (n = 4, *P* < .05 by Student *t* test). We saw clear reduction of GSIS in shATGL-treated pseudoislets as we reported previously (Fig. 3B and 3C) (6). NG497 reduced GSIS in some donors but did not reach statistical significance after 4 donor pseudoislets were tested. Insulin contents at the end of perifusion did not differ between 3 groups (data not shown). Perifusion of human intact islets from additional donors (total 7 donors) revealed mild reduction of GSIS by NG497 exposure with more prominent change in the first phase of insulin secretion than the second phase (Fig. 3D-3F). The effect of NG497 on KCl stimulated insulin secretion varied depending on donors, some showing reduction but not all (Fig. 3D and 3G). NG497 did not change insulin contents at the end of perifusion (Fig. 3H). To test whether small effect of NG497 on GSIS is due to low availability of FA in perifusion buffer, insulin secretion at 16.8 mM glucose was also measured in the presence of 0.24 mM FA after preincubation with 0.24 mM FA. For 5 donors tested, NG497 did not reduce GSIS significantly compared with control (Fig. 3I).

### Acute ATGL Inhibition Dysregulated Glucagon Secretion From Human Islets

**Figure 4.**
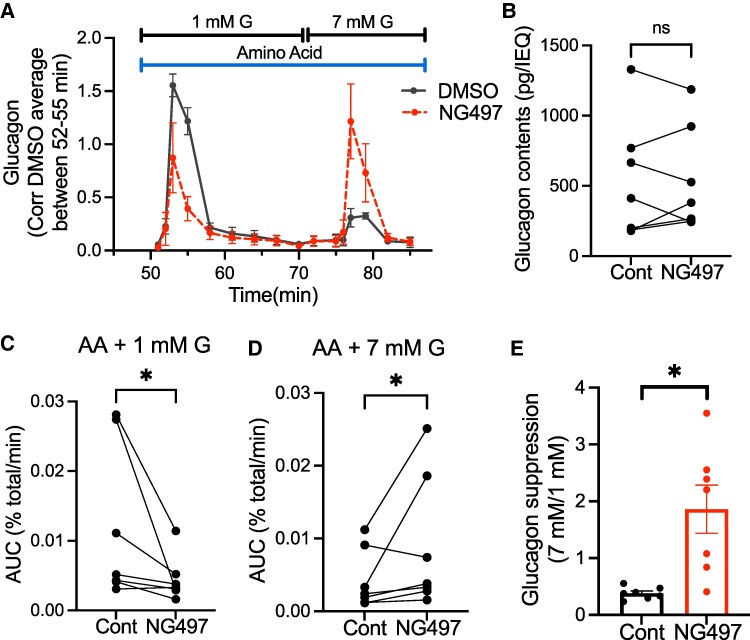
ATGL inhibition by NG497 dysregulates glucagon secretion from human islets. (A-E) Human islets were first perifused by 3.3 mM glucose KRB without amino acid mixture (AA). At the indicated time, perifusion buffer was changed to KRB containing AA and 1 mM glucose (G) followed by 7 mM G. 0.05% DMSO or 40 μM NG497 was present throughout the perifusion from time 0. (A) Perifusion profile combining 5 donors. Glucagon secretion was first calculated as % total. Then, DMSO- and NG497-treated data from each donor was expressed taking average of time 52 to 55 for DMSO treated islets as 1. (B) Glucagon contents per 1 islet equivalent (IEQ). Values from the same donor are connected by a line. (C, D) Area under the curve (AUC) of glucagon peak response during (C) 1- glucose and (D) 7-mM glucose was corrected for glucagon contents and divided by the length of peak (min). (E) The ratio of (D) over (C) as the suppression of glucagon by glucose. n = 7 donors for (B-E). Data are mean ± SEM. *P < .05 by paired Student *t*-test.

Although glucose is a key secretagogue to assess β-cell function (25), amino acids including glutamine, alanine, and arginine are considered to be critical secretagogues for glucagon secretion (21, 24, 26). When control human islets were exposed to amino acid mixture at 1 mM glucose, glucagon secretion increased markedly followed by low sustained secretion (Fig. 4A DMSO, also see Supplementary Fig. S1 (22)), the pattern previously reported for pancreatic islets exposed to glutamine, arginine, and alanine (27-29). Subsequent exposure to 7 mM glucose in continued presence of amino acids resulted in a small peak of glucagon secretion in control human islets (Fig. 4A). Perifusion in the presence of NG497 did not alter glucagon contents at the end of perifusion (Fig. 4B). Notably, NG497 blunted glucagon secretion significantly at the initial exposure to amino acid mixture plus 1 mM glucose (Fig. 4A and 4C). In contrast, glucagon secretion when islets were exposed to amino acid mixture plus 7 mM glucose was higher in the presence of NG497 than DMSO control (Fig. 4A and 4D). With the opposite direction of effect, the ratio of glucagon secretion during 7 mM glucose over 1 mM glucose was significantly higher in NG497-exposed human islets compared with control (Fig. 4E).

## Discussion

Because glucose-stimulated lipolysis by ATGL is reported to be blunted in human islets from donors with type 2 diabetes, it is important to understand the mechanism by which ATGL regulates GSIS in human islets (6). Direct actions of lipolytic metabolites on the insulin secretory pathway (FA on GPR40, 1-MAG on MUNC13-1), transcriptional activation through PPARδ, and protein stabilization by palmitoylation (STX1a) are mechanisms proposed based on genetic downregulation of ATGL in β-cell models (4-6). In the present study, we aimed to gauge the impact of acute vs chronic ATGL inhibition on GSIS in human β cells using a human-specific ATGL inhibitor, NG497 (12). NG497 inhibits GSIS mildly, primarily during the first phase in human islets, an extent that is somewhat smaller than one elicited by shRNA mediated downregulation of ATGL in human pseudoislets (Fig. 3) (6). Thus, chronic targets such as PPARδ and protein palmitoylation may predominantly contribute to impaired GSIS after ATGL downregulation in β cells. This is in line with statistically significant but limited recovery of GSIS by the supplementation of 1-MAG in ATGL downregulated human pseudoislets (6). As perifusion constantly removes FA released by lipolysis, the augmentation of FA through GPR40 may be less prominent and other intracellular targets of lipolysis may be more highlighted in perifusion. Also, FA and 1-MAG generated from diacylglycerides by hormone-sensitive lipase may be sufficient to support GSIS acutely.

Glucagon secretion is reported to be dysregulated in both type 1 and type 2 diabetes (20, 30). The current study demonstrated functional significance of ATGL in glucagon secretion from human islets that appears to be modified by glucose concentration. Under 11 mM glucose culture, LD accumulation after ATGL inhibition was present but less prominent in α cells compared with β cells indicating that ATGL activity is lower in α cells at this condition. However, ATGL avidly contributes to LD degradation in α cells cultured in medium that simulates fasting (LoG + FA). The impact of ATGL inhibition on amino acid-stimulated glucagon secretion differs depending on glucose levels as well. Glucagon secretion in response to 1 mM glucose plus amino acid mixture was lower in human islets exposed to an ATGL inhibitor, but subsequent glucagon secretion under 7 mM glucose was higher in those treated by NG497. Interestingly, FA, metabolites generated by ATGL, have been reported to modulate glucagon secretion in both directions. FA are reported to increase glucagon secretion by raising cytosolic calcium in rat and mouse islets (31, 32), a process that may be partially mediated by FA activation of GPR40 cell surface receptor (33). More recently, it is proposed that β oxidation of FA supports glucagon secretion under low glucose condition and the suppression of β oxidation under high-glucose conditions reduces glucagon secretion. The mechanism proposed is that the increased availability of glucose switches energy production to the tricarboxylic acid cycle fueled by glucose and reduces β oxidation of FA, resulting in lower ATP production at high glucose concentration in α cells (10, 11). On the other hand, it is proposed that palmitoylation of the K_ATP_ channel by FA increases the opening of K_ATP_ channel in a mechanism separate from the regulation of K_ATP_ channel closure by ATP/ADP level and reduces glucagon secretion (34). FA released by ATGL have been shown to contribute to β-cell oxidation (1), the activation of GPR40 receptor (2), and protein palmitoylation (6) in non-α cells. Thus, future studies should address whether ATGL inhibition affects the generation of FA, GPR40 receptor activation, FA oxidation, and palmitoylation of K_ATP_ channel at different glucose levels in alpha cells.

The current study has some limitations. Considering that amino acids are the key regulator of glucagon secretion in vivo (35, 36), glucagon secretion assay by perifusion is increasingly performed in the presence of amino acid mixture (19, 20, 37, 38) or tests response to amino acids (39, 40). Importantly, 2 studies performed in the presence of amino acid mixture demonstrated defects in islets/pancreas slice from type 1 diabetic donors (20, 38). However, compared with glucose-centered regulation of insulin secretion, the regulation of glucagon secretion by nutrients is multifactorial and complex (24, 41). Thus, the optimum combination and concentration of amino acids that elucidate the regulation of glucagon secretion under pathophysiology is yet to be established. We need to bear in mind that ∼mM concentration of amino acids often used is clearly higher than circulatory levels of low abundance amino acids, such as arginine (36). Nevertheless, NG497 has highlighted that lipolytic metabolites including FA may impact glucagon secretion along with glucose and amino acids. Also, we do not know what drives a small second peak of glucagon secretion in control islets when glucose is switched under continued presence of amino acid mixtures (Fig. 4A). It requires future studies to address how the switch in glucose affects factors regulating glucagon secretion such as ion channel activity and ATP production in α cells. Human islets show great heterogeneity, and acute change in lipolysis may be important in some but not all islets, especially for the second phase of GSIS, KCl response, and FA response that varied among donors (Fig. 3F, 3G, and 3I). It is also important to note that human islets are cultured in medium containing less lipids than in circulation. Combined with the effect of amino acids, it will be important to assess the contribution of ATGL-mediated lipolysis in human islets in an environment close to in vivo milieu in the future.

In summary, the current study indicates that ATGL-mediated lipolysis is an active process in both human β and α cells and plays a critical role for the regulation of both insulin and glucagon secretion in human islets.

## Data Availability

Some data sets generated during and/or analyzed during this study are not publicly available but are available from the corresponding author on reasonable request.

## Abbreviations

ATGL: adipose triglyceride lipase
FA: fatty acid
GCG: glucagon
GSIS: glucose-stimulated insulin secretion
INS: insulin
KRB: Krebs-Ringer bicarbonate buffer
LD: liquid droplet
PPARδ: peroxisome proliferator-activated receptor δ
SEM: standard error of the mean
STX1a: syntaxin1a
TG: triglyceride
1-MAG: 1-monoacylglyceride
